# Modification of childcare’s outdoor setting for toddler physical activity and nature-based play: A mixed methods study

**DOI:** 10.1371/journal.pone.0309113

**Published:** 2024-09-20

**Authors:** Chelsea L. Kracht, Amanda E. Staiano, Melissa Harris, Kristin Domangue, Michelle Grantham-Caston

**Affiliations:** 1 Population and Public Health Division, Pennington Biomedical Research Center, Baton Rouge, Louisiana, United States of America; 2 Division of Physical Activity and Weight Management, Department of Internal Medicine, University of Kansas Medical Center, Kansas City, Kansas, United States of America; 3 Louisiana State University, Baton Rouge, Louisiana, United States of America; University of Connecticut Health Center: UConn Health, UNITED STATES OF AMERICA

## Abstract

**Background:**

Toddler physical activity is critical for child health, but little is known about its applications in early childhood education (ECE) centers. The aims of this study were 1) to determine if pragmatic and nature-based modifications to an ECE center’s outdoor setting were feasible and acceptable, and 2) to identify barriers and facilitators of toddler physical activity at ECE centers.

**Methods:**

A multiphase mixed-methods study (QUANT+QUAL) was conducted. In the QUANT study, a stepped, sequential modification of the outdoor setting, using pragmatic and cost-effective nature-based elements, was conducted with a delayed control group over 10-weeks, with follow-up assessments 3-months later (week-20). Five elements (tree cookies, garden, teacher training, playground stencils, and nature table) were introduced individually. Feasibility was assessed using teacher surveys. Acceptability and engagement were assessed by direct observation of toddler use of outdoor elements. Accelerometers were used to assess toddlers’ physical activity during outdoor sessions. The QUAL study included semi-structured interviews from ECE center directors (*n* = 27, 55.6% African American, 92.5% female) that were analyzed using content analysis for themes related to 1) toddler physical activity, 2) barriers and facilitators to toddler outdoor environment changes, 3) perspectives on nature-based elements, and 4) provider training. Member check focus groups (*n* = 2, 7-10/group) were conducted for additional interpretation. All three sources were reviewed for integration.

**Results:**

Toddlers engaged in physical activity for most of the outdoor sessions (>85%). This limited the effect of the intervention, but aligned with directors’ and members’ sentiments that toddlers were already quite active. Across directors, reported barriers to toddler outdoor environment changes were teacher turnover and finances. All nature-based elements, except the stencils, were feasible and acceptable, even at week-20. Directors and members identified additional age-appropriate elements, and desired pragmatic training with technical assistance and funding to implement these changes. Directors and members also desired a curriculum that could be used outdoors.

**Conclusions:**

Nature-based elements were feasible and acceptable to toddlers. Directors were enthusiastic about nature-based elements, but barriers exist in linking directors with these elements. Opportunities to combine toddler-age curricula and pragmatic options for outdoor play may improve ECE centers’ outdoor settings and ultimately toddler health.

## Introduction

Physical activity is a key component of children’s physical, social, and cognitive development [1–3]. Even as early as 1–2 years of age (toddlerhood), inadequate physical activity may result in excess weight gain and further adverse health consequences, such as childhood obesity [4, 5]. When children spend time outdoors, they are more likely to be physically active and engage in experiences that promote social, emotional, and motor development [6, 7]. Based on the socio-ecological model of influence, the amount of time children spend outdoors is impacted by their individual, parental, and home environments [8]; for example, girls and children who are racial or ethnic minorities may spend less time outdoors compared to their counterparts [8]. Another important environment is the formal school or preschool setting [8, 9], which may support continued outdoor time for long-term benefit.

Early childhood education (ECE) settings are ideal for the promotion of outdoor time and physical activity, as the majority of toddlers spend their waking weekday hours attending these settings [10]. Preschooler fundamental motor skill programs, which seek to promote later physical activity, frequently incorporate curriculum, and indoor activities, and conduct lessons during required outdoor time [11, 12]. Promoting physical activity in ECE settings with curriculum and indoor activities may be difficult, however, due to varying preferences and requirements. In addition to employing fundamental motor skills program lessons, ECE settings also promote preschooler physical activity while outdoors with physical [12, 13] and/or social environment changes (e.g., teacher training) [12, 14], as most ECE settings do not meet best practices for either of these areas [15, 16]. Still, these approaches report mixed effects [12–14, 17]. Because most of the evidence from ECE settings focuses on preschoolers, it is unclear if toddlers, who may have engaged in social activity differently and have less developed motor skills, would also benefit from these approaches. All reviews that have investigated improving young child physical activity in ECE settings found few or no studies focused on toddlers (ages 1–2 years) [12–14, 17]. Moreover, a review focusing specifically on infants’ and toddlers’ device-based physical activity measurements found no studies that were conducted in ECE settings and suggested this as an area for future research [18]. Identifying feasible outdoor modifications and appropriate teacher training for toddlers may help promote outdoor time and physical activity in ECE settings.

Emerging evidence suggests that nature-based elements in ECE settings’ outdoor environments may be a pragmatic option to promote child physical activity and cognitive development [19–21]. Nature-based elements may promote nature-based play, or outdoor play in natural environments like shrubs or gardens; this would, in turn, promote more physical activity and social play [22]. A cross-sectional study in 22 Dutch ECE centers found that, in toddlers specifically, nature-based elements were positively associated with overall toddler physical activity, especially among toddlers with anxiety and who were overweight [23]. Continued engagement and support of these changes may necessitate teacher training [24] and consideration of structure and organization within the ECE setting [24].

To fill this gap, we examined whether age-appropriate nature-based elements were feasible and acceptable to toddlers and identified barriers and facilitators to translating nature-based elements into a variety of ECE settings. We conducted a mixed methods study, with quantitative (pilot study) and qualitative (semi-structured interviews) studies in parallel. The quantitative portion was a pragmatic, quasi-experimental pilot study; it examined changes in toddler physical activity through sequential modification of nature-based items to an ECE setting’s outdoor environment. The qualitative portion was semi-structured interviews; they identified barriers and facilitators of toddler outdoor play and nature-based modifications in various ECE settings. These results may help develop nature-based elements for toddlers and respective teacher training for translation into a range of ECE settings.

## Methods

### Overall study

We conducted a multiphase, mixed methods study: a non-randomized pilot study that added nature-based elements to promote outdoor play in two separate ECE centers (QUANT), and semi-structured interviews with ECE directors (QUAL). We used a parallel sampling approach, where the samples for the QUANT and QUAL components were different, but from the same population region [25]. This population was ECE centers that serve toddlers (ages 1–2 years) within a specific southern U.S. state. This study follows the mixed methods reporting standards (see **S1 Table in S1 File**) [26, 27]. These studies were conducted as part of a community (author, m-PI: MGC)-academic (author, m-PI: CLK) partnership; a local community advisory board reviewed study design and provided input on community involvement and conduct prior to commencement. The qualitative and quantitative studies recruited and collected data between August 2021-March 2022. For additional interpretation, focus groups for quantitative study sites were conducted between March-August 2022. All studies were approved by the Pennington Biomedical Research Center Institutional Review Board.

### QUANT: Pilot study

#### Study design

A non-randomized stepped within-subjects design with a wait-list control was conducted to examine the feasibility, acceptability, and preliminary effectiveness of social and physical outdoor environment changes on toddler physical activity. ECE center eligibility criteria included: licensed in a specific southern U.S. state, served ≥10 toddlers (ages 1–2 years), and had an outdoor environment specific to toddlers (i.e., no mixed ages in outdoor environment). The study was initially designed as a single site intervention, but after obtaining additional grant funding, we sought another center to serve as a wait-list control group. To ensure comparable amounts of existing nature based elements, centers were matched based on their Play and Learning Settings score from the Preschool Outdoor Environment Measurement Scale (POEMS) [28]. POEMS is an observation-based measure of ECE social and physical environment incorporating nature-based elements (range: 0–100%), with a higher score indicating more nature-based elements incorporated into their outdoor environment.

Directors provided written consent before procedures, and reported current outdoor play best practices based on the widely used and validated Nutrition and Physical Activity Self-Assessment for Child-Care Survey (NAPSACC) tool. The NAPSACC tool assesses four components of outdoor play (time, environment, policies, and training) and has been used as part of a curriculum and a technical assistance program (GoNAPSACC) that seeks to improve these components [29]. In this study, the NAPSACC tool was used at baseline to evaluate existing best practices, and a curriculum based on the GoNAPSACC program was used as a nature-based element. Toddlers who planned to attend the center for the next 12 weeks based on parental report were eligible. Director written consent was sought for participation in this study and viewing outdoor play areas. Teacher written consent was sought for baseline surveys and feasibility surveys (intervention only). Parents gave written consent for their toddler to participate and completed a demographic questionnaire at baseline. The lead teacher for each site received $100 USD compensation for their participation, which was not based on the number of children who participated. Parents received small toys for completing surveys (~$5 USD total).

#### Intervention

Outdoor setting modifications were chosen to be cost-effective (<$1500 USD total) to match state-provided grants. Modifications included a variety of play elements: fixed play equipment (tire garden, playground stencils, and nature tables), portable play equipment (wooden stepping discs, or “tree cookies”), as well as teacher training. Elements were positioned based on previous evidence of outdoor area use [30], and agreed upon by the ECE director. For initial feedback on placement and implementation, a beta-test was conducted prior to measures at an ECE center with a similar Play and Learning Setting scores (46.1%) to intervention (53.8%) and control centers (46.1%).

#### Measurements

The intervention lead teacher reported element feasibility one week after implementation of items, and overall element feasibility at the end of study. The element feasibility survey had two questions: 1) how easy or difficult was it for the children to play with the item, and 2) how likely were the children to play or engage with the item. The overall element feasibility survey had four questions: 1) how children felt about the elements overall, 2) the success of the elements for children to be physically active, 3) success of elements for children to explore nature, and 4) success of the elements to help children with their social and emotional skills. All questions included a 5-point Likert scale response. The overall element feasibility score was the average of these four questions. The lead teacher also provided written comments at the end of these surveys.

Acceptability and engagement in elements was measured using the Scoring for Outdoor Physical Activity in Youth, which has previously been used in centers [31]. Video cameras recorded outdoor sessions (~2 sessions/day, ~30 minutes/session, total: ~60 minutes/day) and elements used (tree cookies, garden, stencil, and table). Scans were conducted every 60 seconds to classify the number of toddlers using an outdoor area/element. Staff completed training and obtained >90% agreement prior to measurement. Acceptability was defined as the maximum number of toddlers using an element at one time as a percent of the total toddlers, and engagement was defined as the amount of time an element was used during the outdoor session as a percent of the total session. Both factors were defined as percentages rather than numbers, as sessions varied in the number of toddlers (range: 7–11) and time outdoors (range: 21–32 minutes/session). The primary effectiveness outcome was percent time engaged in any level of physical activity during available sessions, also referred to as total physical activity (TPA). TPA was chosen as the primary outcome since available international guidelines for physical activity in toddlers (ages 1–2 years) focuses on accruing at least 180 minutes/day of TPA for health benefit [32]. TPA is defined as the sum of light and moderate-to-vigorous physical activity (MVPA). Physical activity intensity was measured using ankle-worn accelerometers (ActiGraph GT3X, Pensacola, FL) during both morning and afternoon outdoor sessions. Accelerometers recorded in 15 second epochs, and counts were compared to physical activity intensity standards for ankle-worn accelerometers in toddlers [33].

Exploratory outcomes were changes in toddler’s connectedness to nature and psychosocial wellbeing. Parents completed two questionnaires at baseline and week-10 related to connectedness to nature (Connectedness to Nature Inventory [CNI], 16 questions) and toddler psychosocial wellbeing (Strengths and Difficulties Questionnaire [SDQ], 25 questions total) [34, 35]. Both questionnaires are validated in older children (3–5 years), and ask questions related to children’s habitual behaviors using Likert Scale responses [34, 35]. The CNI includes four subdomains (enjoyment in nature, empathy for nature, responsibility toward nature, and awareness of nature), where a higher score indicates a more frequently exhibited behavior. A higher Total Difficulties score from the SDQ also indicates more difficulties, with ranges from 0–40.

#### Intervention procedure

For the intervention group, baseline measurements (director and teacher surveys, parent surveys, anthropometrics, video, accelerometers) were completed (week-0), then the first element was added to the outdoor setting (week 1), and then toddler physical activity and engagement (videos and accelerometers) and a lead teacher survey were conducted (week 2, See **Table 1**). When each element was installed, the lead teacher received materials that described its use and maintenance (for example, ideas on how to use nature table, and upkeep of table). This cycle was then repeated with each element. The 1 week delay reduced novelty effects. The order prioritized nature-based elements (tree cookies and tire garden) without confounding teacher training. Stencils were added last, since a separate pilot study found preschoolers improved their fundamental motor skills after these stencils were added to their ECE center [36]. The engagement in the nature table was assessed 3 months later (~week 20) due to shipping delays.

**Table 1:** Stepped wedge within-subjects diagram.

			Week of Intervention												
Nature-based element	Cost (USD)	Description	0	1	2	3	4	5	6	7	8	9	10	19	20
Portable play equipment: tree cookies	$175	Non-permanent and durable stepping discs, put onto existing grass to guide child movement			***x***		***x***		***x***		***x***		***x***		***x***
Fixed play equipment: tire garden	$350	Planter flowers (herbs, ornamental plants, and vegetables that were non-toxic and allergen free) in a tire set for children to explore					***x***		***x***		***x***		***x***		***x***
Staff training: curriculum on outdoor play	$20	Physical binder and virtual information on facilitating two structured activities and promoting toddler active play based on existing GoNAPSACC outdoor play curriculum							***x***		***x***		***x***		***x***
Fixed play equipment: sidewalk stencils	$325	Sidewalk stencils to create a path of movement based on engagement with other children in gross motor activities, made with spray chalk									***x***		***x***		***x***
Fixed play equipment: nature table	$620	Nature table (Play With a Purpose, Owatonna, MN), toddler microscopes, and nature-based items with a table for interaction, fostering curiosity in nature and free play													***x***

X = indicates assessment of element use; shaded region indicates time during which element was installed within the outdoor environment; GoNAPSACC = Nutrition and Physical Activity Self-Assessment for Child-Care Training program.

#### Waitlist control procedure

For the waitlist control group, baseline assessments were conducted in a similar manner to the intervention group: the director and lead teacher completed surveys, parents completed child-related surveys, toddler anthropometrics were collected, and toddler physical activity was measured through devices and video recording. The site was instructed to not modify their outdoor play area for toddlers, and no changes were made between baseline and follow-up. At follow-up (week-10), the child procedures were completed, and parents completed surveys. The natural elements were then implemented in the control site after the follow-up visit. Baseline (week-0) and follow-up (week-10) were conducted during the same weeks as the intervention group’s respective assessments to control for any seasonality in child physical activity.

### QUAL: Semi-structured interviews

Eligible participants included directors and owners (herein: directors) of center-based ECE settings, specifically licensed ECE centers (herein: centers) within the same southern U.S. state who were not participating in the pilot (study 1). The sites participating in study 1 were included in focus groups for their feedback only after both studies were completed (see Overall Study). Providers from home-based ECE settings were not eligible, since these settings have different regulations for outdoor space compared to centers [37, 38]. The sample was based on a state-provided list of all ECE centers. Centers were stratified by federal assistance funding (yes/no) based on the previous year (2019–2020), then randomly invited to participate. We sought to include between 12–15 centers for each federal assistance status. Directors were emailed and contacted via phone to participate. The semi-structured interview was conducted with each individual director by phone, by a researcher trained in qualitative methods (CLK). All interviews were audio recorded and transcribed verbatim.

After confirming eligibility, verbal informed consent was obtained prior to study procedures. Directors reported age, sex, race, education level, and years of service, then answered questions related to outdoor play best practices based on NAPSACC tool (See **S2 Table in S1 File**) [29]. After these questions, directors were asked 1) their perceptions of toddler physical activity, 2) barriers and facilitators to toddler physical activity and outdoor environment upgrades, 3) their interest in nature-based elements, and 4) training on teacher involvement in the outdoor setting. Interviews lasted around 37 minutes (range: 13–64 minutes). For their participation, directors received $25 USD worth of educational supplies and outdoor play handouts.

### Member check and additional interpretation

An initial qualitative and quantitative analysis was completed in February 2022 and presented to the intervention site (March 2022) and control site (August 2022) separately. These focus groups included teachers and directors of the respective sites control sites (*n* = 7-10/group). Feedback shared from these focus groups was used as a check amongst community members, and to provide additional interpretation of our quantitative and qualitative results.

### Data analysis

For QUANT and QUAL samples, NAPSACC responses were scored based on age-specific best practices for time, environment, policy, and training opportunities (met vs. not met) [39, 40]. The number of best practices met was summed for a possible total of 0–14, with a higher score indicating more best practices met.

#### QUANT: Statistical analysis

Items that scored ≥4.0 out of 5.0 on ease/difficulty for use and likeliness for toddlers to engage with elements were deemed feasible. Acceptability and engagement (%) from video recordings were used to qualitatively supplement teacher survey findings. Overall feasibility was the average of four questions (overall acceptability, physical activity, explore nature, and use of social and emotional skills). Differences in demographics between groups were compared using an independent t-test chi-square or Fisher’s exact test. For effectiveness, mixed models with repeated effects were used to examine the effect of time, group, and their interaction (group*time) on TPA and MVPA from week-0 and week-10. Mixed models were also used to examine changes in CNI subdomains and total difficulties score. All analyses were performed using SAS (version 9.4, Cary, N.C.), and significance was set at *p*<0.05.

#### QUAL: Thematic analysis

Saturation was reached (*n* = 27), and no additional interviews were conducted. Transcribed interviews were coded using thematic analysis with deductive reasoning, where four *a priori* major codes were chosen based on the interview guide sections. Two authors (CLK and MGC) who were trained in qualitative analysis reviewed the transcripts to create subcodes for these major codes. A final codebook was created after a second review of transcripts. These reviewers coded transcripts independently and resolved disagreement by discussion. To determine themes, selected codes were read by each reviewer independently using a grounded theory approach, allowing themes to emerge from responses to these questions [41]. The finalized themes were informed by study aims and reviewed one final time. Based on prior literature, an emergent theme was identified when at least 20% of the sample provided relevant comments [42]. All data analysis was performed using Atlast.TI software (Berlin, Germany).

#### Integration

Results from all three parts of this study (the quantitative pilot study, qualitative interviews, and member check focus groups) were integrated to compare meta-inferences. The meta-inferences were divided based on qualitative feedback into the constructs of 1) toddler physical activity and outdoor play, 2) barriers and facilitators to toddler outdoor play and environment changes, 3) perspectives and interest in nature-based elements, and 4) professional development for outdoor play. These results were then combined in a joint display for appropriate guidelines [43].

## Results

### QUANT: Pilot study

Two centers (one university-affiliated, and one center-based) and 15 toddlers participated. The intervention site had one parent decline to participate, thus 10/11 potential toddlers (90%) participated. The control site had more than 10 children available, but only 5 parents consented to their child’s participation. Demographics were not available for toddlers who did not participate. Control toddlers were older than intervention toddlers (31.11 ± 2.57 months vs. 24.5 ± 2.36 months, respectively, *p*<0.01), and there was no difference between groups in child sex, race, Hispanic ethnicity, or household incomes (*p*’s>0.05, **S3 Table in S1 File**). Across centers, most child participants were girls (80%) and parent-identified as White (80%).

At baseline, intervention and control centers were similar in outdoor play best practices met (3 vs. 4) and Play and Learning Setting scores (53.8% vs. 46.1%). At week-10, the intervention center improved its Play and Learning Setting score (76.9%), but the control center had minimal change (53.8%). All elements were rated as feasible on both questions (≥4.0/5.0), except stencils (2/5 for both questions). The teacher reported the toddlers were more interested in manipulating and playing in the chalk that was used to fill in the stencil on the concrete, rather than engaging in the stencil’s patterned walking or jumping activity. The lead teacher reported the tire garden was “neither easy nor difficult” due to toddlers playing in dirt (3/5) at week-4, but the tire garden received high praise through qualitative written feedback at the end of the study. Overall feasibility was high (4.75/5). The lead teacher noted the continued use of the garden and nature table, ease of teacher training, and the tree cookies promoting toddler social and motor skills after week-20.

Many children consistently used the tree cookies, garden, and table, but not the stencil (see **S1 Fig in S1 File**), indicating high acceptability. The stencil was only used by two children after its initial implementation (week-8), and no children used it in the subsequent weeks during the observation periods. The tree cookies had similar acceptability at week-20 (40% of children used) as week-2 (55%), though the garden was used by fewer children (10%). Time spent engaging in tree cookies and garden was variable (range: 0–53% for both), but these elements were still used during each session, even at week-20. The teacher led a structured activity from the teacher training during the week-6 morning play session, which may explain lower engagement with the tree cookies and garden. Considering play sessions after implementation, the tree cookies (11-sessions), garden (9-sessions), stencil (6-sessions), and the table (2-sessions) were used 22.7%, 21.8%, 2%, and 78% of the total time while outdoors, respectively. The lead teacher was only present at one afternoon session (week-2), which may contribute to slightly lower acceptability and engagement in afternoon sessions. The afternoon sessions were conducted by teachers who were unaware of the primary outcomes for the study.

Detailed results of the accelerometry measurements for toddler physical activity are found in **S3 Table in S1 File**. For effectiveness, there was an independent effect of group and time on total outdoor time and TPA (*p’*s<0.05 for each group and time). The intervention group had a steady decrease in outdoor time (66.8 ± 7.9 minutes) and TPA (62.3 ± 7.3 minutes) from week-0 to week-10 (outdoor time: 53.0 ± 2.1 minutes, TPA: 48.9 ± 3.5 minutes); the control group started at a higher outdoor time (81.5 ± 1.0 minutes) and TPA (75.4 ± 2.3 minutes) at week-0, which decreased at week-10 (outdoor time: 73.4 ± 0.8 minutes, TPA: 63.9 ± 6.5 minutes). However, when considering a group and time interaction, there was no effect of the intervention on toddler physical activity (*p* = 0.13). There were no other effects of group, time, or their interaction on TPA (%) or MVPA (minutes or %, **S3 Table in S1 File**). Upon further review of toddler physical activity across the intervention (week-0 to week-10), toddlers spent most of their outdoor time in TPA (range: 92.3–97.1%), and most of this activity was MVPA (40–52%, **S2 Fig in S1 File**).

There was a significant group*time effect for empathy for nature score (**S3 Table in S1 File**). The intervention group had a lower score at baseline, which slightly improved across the intervention (week-0: 3.40 ± 0.75, week-10: 3.67 ± 0.82), whereas the control group’s score decreased over time (week-0: 4.53 ± 0.69, week-10: 3.93 ± 0.64). There were no other significant changes in connectedness to nature scores or total difficulties scores across group, time, or their interaction.

### QUAL: Semi-structured interviews

As shown in **S3 Fig in S1 File**, 27 directors completed interviews of 153 initially contacted. Most directors were senior (>10 years in ECE) and met 4 (range: 2–8) outdoor play best practices (**Table 2**). Due to expansion of federal programs during the COVID-19 pandemic, the majority accepted federal funding (66.7%, 17/27) though an even split was planned. As such, the responses from all directors were evaluated together. The major themes included 1) toddler physical activity practices, 2) barriers and facilitators to toddler outdoor environment changes, 3) perspective and interest in nature-based elements, and 4) professional development related to outdoor play. The major themes are presented in detail and with selected illustrative quotes in **Tables 3 and 4**.

**Table 2:** Demographics of ECE directors in aim 2 (*n* = 27).

	Median (range)	n (%)
***ECE Director Characteristics***		
Age, years	50 (26, 78)	
Female		25 (92.5)
Race		
White		11 (40.7)
African American		15 (55.6)
Other		1 (3.7)
Highest Level of Education		
High School		1 (3.7)
Associate’s/Bachelor’s		11 (40.7)
Graduate/Professional		15 (55.6)
ECE service, years	20 (3, 44)	
<5		1 (3.7)
5–10		7 (25.9)
11–20		8 (29.6)
>21		11 (40.7)
***ECE Center Characteristics***		
Setting type		
Center-based (no specifical affiliation)		14 (51.8)
Church-based		5 (18.5)
Early Head Start		5 (18.5)
Montessori		2 (7.4)
University-affiliated		1 (3.7)
Accept Federal funding		17 (66.7)
Enrollment Capacity, children total	75 (19, 281)	
<50		9 (33.3)
50–99		7 (25.9)
100–149		7 (25.9)
>150		4 (14.9)
***Outdoor Play Best Practices Met***		
Outdoor time	1 (0,2)	
60+ minutes/day		17 (66.7)
3+ times/day		3 (11.1)
Environment	3 (1,5)	
Variety (5+) types of equipment		5 (18.5)
Shade: <1/4, or >3/4		3 (11.1)
8+ play areas		3 (11.1)
Large enough for all children		22 (81.4)
Not limited on portable play items		17 (66.7)
Portable play is always available		24 (88.8)
Path for wheeled types, 5+ feet, looped		3 (11.1)
Fruit and vegetable garden		3 (11.1)
5+ types of outdoor activities		0
Policies: 3+ policies related to outdoor play		0
Professional development	0 (0,2)	
3+ topics related to outdoor play		2 (7.4)
2+ times/year		5 (18.5)
Total Outdoor Play Best Practices Met	4 (2,8)	

ECE = Early Childhood Education.

**Table 3:** Thematic findings related to toddler physical activity, outdoor play, and outdoor environment^.

Subthemes:	Quotes representative of subtheme
***Theme 1*: Toddler Physical Activity and Outdoor Play Practices**	
Providers felt toddlers are naturally energetic and moving throughout the day.	• *“Toddlers are moving all the time*. *There’s very*, *very little time that they’re just sitting and looking at something*. *They’re on the go*, *their little legs are taking them about the room and exploring all the time*.*” (Director #11*, *Center-based care)* • *“They go to different centers*. *The kitchen center*, *blocks center*. *The only time our toddlers are actually sitting down is snack time*, *lunch time and if they’re doing a one on one activity with their teacher*.*” (Director #17*, *Church-based care)* • *“I try to keep them moving all the time” (Director #23*, *Center-based care)*
Toddler physical activity mainly occurred in groups and with teachers	• *“Our students are very active*. *Again*, *we’re a Montessori schools have every single learning materials is done with hands on tangible materials*, *so they’re constantly moving*. *In our classroom*, *we even have a large motor area where you know*, *they can work on their bodies*.*” (Director #12*, *Montessori)* • *“I think it’s really all about the teacher and their level of engagement*. *You know*, *and how they encourage the children I think*, *you know*, *kids are going to do you know*, *things that they do regardless*. *But*, *you know*, *teachers are the ones that really facilitate and get them to do more*, *you know*, *when they do go outdoors and things like that” (Director #15*, *Center-based case*, *GoNAPSACC training)* • *The teachers keep them pretty active throughout the day and if they are not outside*, *running around*, *playing on the toys*, *they already in the room moving around*, *they are constantly moving around in the room and they have centers inside of the room that they have and the teachers keep them busy*.*” (Director #22*, *Early Head Start)*
Open space and nice weather were important facilitators for toddler physical activity and outdoor play.	• “*Well I actually think outdoors*, *you know*, *most children love to go outside*. *It’s more area to run… sometimes it’s the weather we get a lot of rain*, *we get*, *you know*, *sometimes it’s too wet*. *Sometimes it may be too cold*, *those sorts of things” (Director #2*, *Early Head Start)* • *“I would have a way bigger yard and more structures for them to climb on and more stations*. *And I would put them a big cement track to ride their bikes on*.*” (Director #25*, *Montessori)* • *“I think a track or toddlers could ride away from the other things are going on in that space*, *would be helpful*. *Because you have the riding toys*, *and you also have the other things going on*. *My toddlers don’t have any swings*, *… I don’t have anything like that*, *because it takes up so much space*. *So*, *I would like to maybe divide it in areas where you have the movables and the non-movables*.*” (Director #11*, *Center-based care)*
***Theme 2*: Barriers and Facilitators to Toddler Outdoor Environment Changes**	
Providers expressed concerns hiring and retaining staff when discussing teacher’s role in toddler development and physical activity.	• *“We’re in a national crisis with employees and it’s hard to keep good employees and it’s hard to find good employees*. *So*, *um*, *to try and train someone*, *this is good*, *but we’re not babysitting*, *we’re childcare and we have to promote a lot of things and one of them has to be how to be more active*. *And I’m doing this to employees who are getting paid*, *not minimum wage*, *but close to it*. *They have to consider who’s willing to work for this high stress paying job that is not getting paid very much money” (Director #10*, *Church-based care)* • *“Right now*, *just having to you know*, *go through the readjustment and redefining processes and procedures and ways of COVID*. *You know*, *try not to exhaust the staff too much with too many new things at one time*.*” (Director #18*, *Early Head Start)* • *“If you have the wrong people in the wrong place*, *if you have the wrong type of people working in the center*, *we*, *you know*, *you can’t just hire somebody with a pulse to work here*. *So*, *I personally*, *in our center*, *we work very*, *very hard to find the right people to work here*. *Because um*, *you know*, *money is an issue*, *and um as far as uh*, *you got*, *you know*, *which where we*, *you know*, *our*, *the culture here is*, *you know*, *we’re not we’re not Los Angeles we’re not New York*, *you know*, *we’re more of a rural type community” (Director #19*, *Center-based care*, *GoNAPSACC training)*
Finances was a major barrier to outdoor environment upgrades.	• *“There’s lots of equipment and materials for outdoor play but they’re so expensive*.*” (Director #1*, *Center-based care)* • *“Money is always the bottom line always work hard doesn’t matter*, *always the bottom line*, *mhm*.*” (Director #9*, *Center-based care)* • *“Money*. *It’s all about the money to be able to do it which will fix it*. *If I had applied for a grant which I did get awarded the grant so we have some stuff on order with Volunteers of American*. *Bring in for the outdoors so that is going to help*.*” (Director #14*, *Center-based case*, *GoNAPSACC training)* • *“If we had the funds*, *I think that I don’t really think there would be too many barriers*. *Honestly*, *I think you know*, *the funds of course are a big part of it*.*” (Director #15*, *Center-based care*, *GoNAPSACC training)* • *“Playground equipment is very*, *very expensive*. *And that play foam padding that 30 by 30 was already $18*,*000 just to have it so and that’s because they have to do the floor*.*” (Director #26*, *Church-based care)* • *“I have added so much equipment like I keep telling people I’m in the wrong business*. *I belong in the playground business because there’s no way that a playground a piece of playground equipment costs $28*,*000 But hey*, *that’s what it costs*.*” (Director #27*, *University-affiliated care)*

^Participant numbers indicate their type of center-based care, and training status; GoNAPSACC = Nutrition and Physical Activity Self-Assessment for Child-Care Training program.

**Table 4:** Thematic findings related to nature-based elements and professional development for outdoor play^.

Subthemes:	Quotes representative of subtheme
**Theme 3: Perspectives and Interest in Nature-based Elements**	
Many did not have nature-based elements in their environment but were incorporating nature into science activities.	• *“I actually had a playground area developed and drawn to scale*. *That was like the little bug sets*. *And they had like the different types of things that they’re able to do*, *like it was a section where they go hop from lily pad to lily pad*, *a section where they were able to just build with the sand and different things*, *but rather than it being things we have to take in*, *setup*, *and break down*. *It was more of a permanent fixture*.*” (Director #4*, *Center-based care*, *GoNAPSACC Training)* • *“personally*, *I like the aesthetic of things being a little bit more natural*. *So like*, *wow*, *the shape fields are beautiful*. *I would love to be able to plant some big trees out there*. *I would love for our equipment to be a little bit more organic*. *Unfortunately*, *when you’re buying commercial equipment*, *it all comes in bright colors*.*” (Director #12*, *Montessori)* • *“before the holidays near the fall*, *teacher did a nature walk in her objective with the children for them to find an insect and talk about the insect*. *So they would walk around the playground and they but look for insects*.*” (Director #18*, *Early Head Start)*
There was great interest in gardening, from planting fruits and vegetables to creating a butterfly garden.	• *“We want a garden*, *we want to*, *we would love to have a butterfly area*.*” (Director #1*, *Center-based care)* • *“We did enact a garden earlier*, *well this is a new school year so last year*, *our children got to enact according*, *but the only thing with that is they don’t keep up with it… that I have a licensed gardener because my husband’s been doing gardening for like 35 years*, *so he actually introduces them to all types of plants*.*” (Director #5*, *Center-based care)* • *“because in the spring*, *the monarch butterflies come and we have three gardens in our courtyard and we plant milkweed to attract the Monarch caterpillars*, *and then they can take we keep the big plastic magnifying glasses outside so they can look for the little bitty caterpillars and then we have the nets that they can bring into their class*.*” (Director #17*, *Church-based care)*
Providers were interested in having a variety of options, including portable and fixed play equipment upgrades.	• *“I think the freedom of choice*. *They come up with some interesting things to do*.*” (Director #4*, *Center-based care*, *GoNAPSACC Training)* • *“More portable*, *toys*, *of course*, *and variety for sure” (Director #6*, *Church-based care)* • *“I want stationary things*, *almost like stations on my playground*.*” (Director #26*, *Church-based care)*
Everyone was interested in nature-based elements, but faced similar barriers as general outdoor play environment upgrades.	• *“Funding for things that are nature*. *We do get some funding but of course*, *you always need more*.*” (Director #2*, *Early Head Start)* • *“I guess same*, *goes back to funding*. *And having ideas I guess*, *resources to see what*, *what my options are” (Director #16*, *Center-based care*, *GoNAPSACC training)* • *“Yes*, *I think the natural the natural base playground design are a lot safer*. *Right now*, *unfortunately*, *the playground is very commercial*. *It’s safe as far as compliance goes*. *But it’s just not having the funds you know or its resources*.* *.* *. *I think the children gravitate more to what looks natural” (Director #18*, *Early Head Start)*
**Theme 4: Professional Development for Outdoor Play**	
Most directors only had a policy about outdoor time for toddlers	• *“I don’t think we have any stated policies as far as Physical activity with the kids other than it being generically expressed to the parents*, *what are we going to be doing during the day with them*.*” (Director #1*, *Center-based care)* • *“I think licensing is like 60 minutes a day that they are supposed to be which we do that*. *I mean*, *I don’t really have any policy other than the 60 minutes*, *according to licensing that we go out for*.*” (Director #14*, *Center-based case*, *NAPSACC training*,*)* • *“They go outside for at least 30 minutes*. *20 minutes or two minutes*. *Because we have to rotate the playground*.*” (Director #21*, *Early Head Start*, *GoNAPSACC Training)*
Some mentioned the outdoors as a classroom, but few mentioned performing actual curriculum outdoors	• *“I try to emphasize that instead of just letting them go run around and play on everything*, *try to have a specific activity that engages them in terms of what they’re learning inside the classroom*.*” (Director #2*, *Early Head Start)* • *“The outside was just for free playing*, *but we’re getting more into the structured playing because we’re trying to bring our classrooms outside as well*. *So we’re getting more into the structure play for outside*.*” (Director #8*, *Center-based care*, *GoNAPSACC training)* • *“So you know the teachers are able to use the curriculum outdoors while the children are playing*, *doing different activities with them*. *do a nature walks*. *You know*, *just anything that the teachers can do is especially following along with the curriculum*, *to allow the kids you know*, *to be outside and explore and encourage that*.*” (Director #15*, *Center-based care*, *GoNAPSACC training)*
Some had participated in a recent outdoor environment initiative, which helped change their outdoor environment	• *“[The training] pointed out some things*, *because like*, *even though some of the things*, *well a lot of the things we were already doing*, *but because we didn’t have a written policy*. *So*, *that opened my eyes so that I need to include that in the parent handbook*.*” (Director #4*, *Center-based care*, *GoNAPSACC Training)* • *“We did [have outdoor policies]*, *but now we are changing [their outdoor policies] that because of NAPSACC” (Director #7*, *Center-based case*, *GoNAPSACC training)* • *“It was a lot of things*, *but how you can make the playground an environment*, *how you can construct the outdoor into a learning environment*. *Before way back*, *they thought our learning environment was only classrooms*. *But no*, *learning environments are also outdoors*. *So one of the things I learned form go NAPSACC when I was doing the training*, *that the outdoor can be used as a learning environment as well as the indoor*.*” (Director #21*, *Early Head Start*, *GoNAPSACC Training)* • *“Besides the training they get from me*, *they haven’t received any purposeful for intentional training*.*” (Director #26*, *Church-based care)*
Important aspects of teacher training were in-person format, continuing education hours, and pragmatic training	• *“I mean*, *my preference will be in person*, *but with this pandemic stuff*, *it really is going to just really be up to us*, *you know*, *making sure that whoever comes in to take the proper precautions” (Director #5*, *Center-based care)* • *“we like one on one or you know face to face learning hands on*. *We do not do zooms well; we do not do computers well because you cannot mop your house and keep your kids and get an effective learning experience*. *Sorry*, *doesn’t work for us like that*.*” (Director #9*, *Center-based care)* • *“We need someone who already have worked in centers and they have tried this stuff already because they would be the ones that would be able to tell us more it about than someone who just read about it and they go “oh well I think this is going to work”*. *But when you are working with people that are already in the center and they tried the stuff and they know the teachers that tried it and it wasn’t too hard for them*, *you know they put effort in things*. *You learn more from people who have already done this stuff*.*” (Director #13*, *Center-based care*, *GoNAPSACC training)*

^Participant numbers indicate their type of center-based care, and training status; GoNAPSACC = Nutrition and Physical Activity Self-Assessment for Child-Care Training program.

#### Toddler physical activity practices

Three main subthemes from toddler physical activity practices were: 1) toddlers were naturally energetic, 2) toddler physical activity occurred with other toddlers and/or teachers, and 3) open space and nice weather were important facilitators to toddler physical activity and outdoor play. Accordingly, most directors referenced toddlers being busy or energetic throughout the day in reference to general physical activity, rather than engaging in intense or gross motor activity. As one director (#13) said, “*They are jumping and running and bouncing and they are on the move constantly*. *Inside and outside*. *They really have a lot of energy*.*”* Some (namely, Montessori directors) mentioned the importance of developing motor skills when describing toddler physical activity. Directors mentioned using classroom centers/stations, music and movement, or climbing equipment to keep toddlers active indoors. Some directors mentioned that having an adjacent gym or open space indoors prevented inclement weather from impacting their toddlers’ daily physical activity. Directors were concerned about a variety of weather types, including heat, rain, and cold, which all prevented the toddlers from going outside.

#### Barriers and facilitators to toddler outdoor environment changes

Two main subthemes related to updating existing outdoor play and environment were: 1) concerns hiring and retaining staff when discussing toddler development and play, and 2) finances were a major barrier. Though not specifically prompted, directors discussed staffing challenges when they discussed the teacher’s role in outdoor play. These concerns were presented as the director’s most proximal issue to address when thinking of this age range in their center, rather than outdoor play or physical activity. Directors discussed that issues stemmed from difficulty finding the “right” person to support this developmental age, few applications for employment, and financial constraints that make it difficult to pay toddler teachers a living wage. Accordingly, finances were the main barrier reported (62%, 17/27). As one director (#11) said, “*it always takes something call money*, *honey*. *I mean*, *really*, *it’s the resources to have it*, *to do it*.” Many directors cited figures of over $10,000 when describing the cost for updating the outdoor environment. Thus, major changes to their outdoor environment in recent years were facilitated by grants, which allowed them to have age-appropriate equipment that meet their safety goals and the class size. One financial facilitator was a recent region-specific GoNAPSACC curriculum that provided funds for outdoor portable play equipment, which 25.9% (7/27) of the sample participated in. Even with grants or the GoNAPSACC curriculum, most directors still desired large-scale changes, such as a fixed play structure, more space, shade/canopy, surface changes, or a garden, each of which would require additional funds. As one director (#13) said, “*I would get more types of play equipment for that age group and have probably a larger area that is designated just for them*.*”* Few directors mentioned administrative barriers, such as approval from preschool board.

#### Perspective and interest in nature-based elements

Four subthemes arose from directors’ perspectives and interest in nature-based elements: 1) use of nature in existing activities, 2) excitement for gardening, 3) importance of having a variety of options, and 4) interest in nature-based elements. Most directors did not recognize the term “nature-based” immediately. After given an explanation, some directors mentioned they used sand tables, play structures that resembled outdoor items like bugs or trees, or tree cookies. Nature walks, a facilitated walk to recognize and enjoy nature, were the main nature-based activities performed. Though gardening is not nature-based *per-se*, there was genuine interest in toddlers being connected to nature through planting fruits and vegetables, and butterfly gardens. Gardens were usually described along with science activities, facilitated through outside help of extension specialist, parent, or enthusiastic teachers, but with concerns for safety in this age range. For nature-based upgrades, directors felt that having freedom and various options were important so that toddlers could experiment outdoors and engage in the elements. Barriers to nature-based upgrades were mainly finances or available space. Some directors had already started investigating nature-based items, but were unable to find them or faced financial barriers. These barriers persisted for older age ranges, and were not specific to toddlers. Directors who had not investigated nature-based elements were interested in technical assistance to purchase appropriate items for their environment.

#### Professional development on strategies for outdoor play

Four main subthemes for professional development were: 1) most directors had only a policy about outdoor time and little other guidance, 2) treatment of the outdoors as a classroom, 3) participation in a recent outdoor initiative, and 4) desire for in-person and pragmatic training. First, most directors only had a policy about amount of time toddlers spent outdoors, and no other major guidance for teachers related to physical activity or outdoor play. Many directors mentioned that their policies followed state licensing standards with some adjustments due to remaining COVID-19 protocols. Second, directors felt that the outdoors should be an extension of the classroom, but none were performing curriculum consistently outdoors or had received training on this topic. Third, some directors had participated in a regional initiative that tied outdoor education training with receipt of funds (25.9%, 7/27). The interviewers were not aware of this region-specific GoNAPSACC training program prior to the interviews. Directors enjoyed this model of professional development and the opportunity to obtain funds to update the environment based on lessons learned. Those who participated in GoNAPSACC were more knowledgeable on active play and outdoor environment upgrades. Those without this training drew from their own experiences and licensing guidance. As stated by one director (#17) about their outdoor play training, “*None*, *it’s just informally passed down*.” Finally, if given the option, many directors preferred multiple in-person training courses, as they felt teachers may not be engaged in a virtual format. They wanted an experienced professional to provide training on child-tested options for toddlers to be active, for these classes to count for continuing education credit, and for these classes to be state accredited. The directors stressed the importance of someone seeing their outdoor environment and providing tailored advice for any environmental upgrades.

### COVID-19 pandemic impact on results

All three studies were impacted by COVID-19 restrictions. The quantitative study was impacted in three ways: recruitment, element timing, and teacher retention. Recruitment occurred via teachers and handouts rather than in-person at high traffic times at the ECE center. In-person procedures were also limited for following up on parent survey completion, so parents were emailed to complete surveys or provided a link. Lack of in-person options limited recruitment in the control center and reduced completeness of parent surveys, resulting in missing questionnaire data for two toddlers at week-0 and one toddler at week-10. Second, the timing of elements was impacted by shipping delays, thus this original 12-week study was expanded to a 20-week study to allow for evaluation of the nature table, which arrived 3 months later than expected. Finally, the control center had a new director and lead teacher at week-20, as the original lead teacher switched classrooms; this delayed implementing the center-specific items for the control group and planning member check focus groups.

The qualitative interviews were impacted by limited recruitment. Three centers from original selection (3/153) were out of business, and some centers reported that they were not interested since they were too busy covering staff outages (18/37 contacted, **S3 Fig in S1 File**). All qualitative interviews were conducted via phone as planned to increase participation regardless of the COVID-19 pandemic. Finally, member check focus groups were conducted virtually due to ongoing virus concerns.

### Integration

As shown in **Table 5**, the four main constructs were thoroughly explored through all three studies. First, all three groups found that toddlers were already active, which may have limited the effectiveness of the intervention. The intervention improved the play and learning score–this result aligned with members’ thoughts that “anything helps”, as there is a dearth of options in this age group, and members were still interested in nature-based items. Second, quantitative and qualitative results revealed the unexpected barrier of teacher turnover. This sentiment was not directly discussed in the member check focus groups, but reducing teacher load was discussed in relation to the potential time and burden for upkeep of nature-based elements. The other barrier revealed in the quantitative and qualitative studies was finances. To overcome financial barriers, member check groups suggested using outside collaboration or parent support. Third, members thought most of the natural elements were feasible and acceptable, and there was great interest in gardening and age-appropriate options. Finally, all three groups indicated an interest in pragmatic teacher training and enjoyed the GoNAPSACC training model. The qualitative interviews and member check were also interested in curriculums that could integrate easily with the outdoor environment.

**Table 5:** Joint display of the key findings of the quantitative pilot study, qualitative semi-structured interviews, and member check focus groups.

Construct	Quantitative Pilot Study	Qualitative Semi-Structured Interviews	Member Check Focus Groups	Meta-Inference
**Toddler Physical Activity and Outdoor Play Practices**	• Toddlers engaged in physical activity for the majority (>90%) of their outdoor play sessions, limiting the effectiveness of the intervention. • The intervention improved the intervention center’s play and learning score. • 1 of the 12 outdoor play sessions was cancelled for weather.	• Directors felt toddlers were already naturally active, and activity mainly occurred in groups with teachers. • Open space and nice weather were important facilitators to outdoor play.	• Members agreed that toddlers were naturally active and were not surprised by the toddlers’ high physical activity. • Members felt that “anything helps” regarding toddlers engaging outdoors, despite the intervention’s limited effectiveness. • Members also mentioned open space and nice weather were important for toddler physical activity.	• Toddlers are quite active outdoors, which may preclude any outdoor intervention. • Weather and space are important components of outdoor play.
**Barriers and Facilitators to Toddler Outdoor Environment Changes**	• Director and teacher turnover at control site was a major barrier. • The cost-effective package of natural elements was feasible.	• Directors expressed concern about hiring teachers to facilitate toddler physical activity and development. • Finances were the major barrier.	• Members mentioned opportunities for parents or grandparents to support the elements to reduce cost and long-term adoption.	• Hiring and retaining teachers is an important part of facilitating toddler development and activity. • Finances are a major barrier, potentially facilitated through outside collaboration or parent support.
**Perspectives and Interest in Nature-Based Items**	• 3/4 nature-based fixed and portable play items were feasible and acceptable.	• Many did not have existing nature-based elements and were very interested in gardening. • Providers were interested in having a variety of age-appropriate options (portable, fixed). • Environmental considerations and finances were still barriers.	• Members provided suggestions for garden upgrades, creating small tasks for toddlers to help with garden upkeep, and musical instruments outside. • Members emphasized age-appropriate considerations for toddlers.	• Three nature-based elements were identified as feasible and acceptable, though other options may be available. • There is great interest in gardening as another nature-based activity.
**Professional Development on Outdoor Play**	• The teacher training was feasible and acceptable. • This training included a physical binder and electronic information based on GoNAPSACC.	• Many only had a policy for outdoor play time, and no other guidance. • Directors liked to treat the outdoors as a classroom. • A regional specific GoNAPSACC helped increase provider knowledge and provided funds. • Directors desired pragmatic training.	• Members were also interested in bridging in-class learning to the outdoor area. • Members desired pragmatic training.	• Pragmatic in-person training is desired. • The GoNAPSACC model of training and receiving funds is helpful to those who receive it. • Creating a curriculum that can go outdoors may help facilitate toddlers’ learning and directors’ interest in treating the outdoors like a classroom.

GoNAPSACC = Nutrition and Physical Activity Self-Assessment for Child-Care Training program.

## Discussion

The purpose of this study was to examine the feasibility and acceptability of nature-based modifications to ECE centers that promote toddler physical activity, and to identify barriers and facilitators to their implementation. Our pilot was intended to be cost-effective and pragmatic, and most elements were found to be feasible and acceptable, even 3 months later. Barriers to modifying the outdoor environment were mainly financial, though there were concerns about teacher retention when discussing the toddler age range. A recently provided evidence-based curriculum with grant funds was a facilitator to outdoor environment changes. Greater infrastructure, finance, and training support are needed to enact nature-based elements for toddlers in ECE environments.

In this study, directors felt that toddlers were already active, and that toddlers spent most of their time in physical activity while outdoors. It is well documented that directors, teachers, and parents perceive preschoolers as active, and often note that children are busy rather than engaging in MVPA (e.g., fast walking) [44, 45]. Indeed, many preschoolers meet the TPA guideline for their age [46], but a systematic review did find that only ~40% of preschoolers’ outdoor time is spent in total physical activity [9]. This contrasts with the current study’s findings that toddlers were in TPA for most of their outdoor session (range: 92.3–97.5%); this may have hindered our ability to detect a change resulting from the intervention. The high TPA may be due to their constant movement during the outdoor session, and others have reported that toddlers engage in high TPA across the day (~246 minutes/day) but with a low amount in MVPA (60.16 minutes/day) [18]. As toddlers were often engaged in gardening and with the nature-table, this additional activity in the upper body (e.g., digging with hands) may not have been measured by ankle worn accelerometry. It is possible that physical activity while using elements (e.g., walking around garden) was like activity prior to installment (e.g., walking around yard). The current study used methodology appropriate for this age range, but universal and consistent assessment of physical activity in toddlerhood is still emerging [18].

The directors emphasized the importance of high-quality teachers and teacher retention to support toddler development, outdoor play, and physical activity efforts. This qualitative finding was reflected in the quantitative pilot study: within 6 months, the control site had a new director, and the lead teacher had switched classrooms. Following the COVID-19 pandemic, ECE centers have faced challenges retaining staff [47], given their status as essential workers, low pay, and high stress [48]. Staff are critical to facilitating child health behavior changes in the ECE setting [49]. This barrier speaks to greater changes and support needed within facilities and macrosystems for implementing any program.

The cost-effective program was feasible and acceptable, but many directors desired larger modifications for their environment. The amount chosen for the pilot study was based on state grants in this U.S. state, which are lower in amount ($1500 USD) than that proposed by the directors (>$10K USD). It may be expected that finances are a major barrier, as a significant amount of money is required to make these larger modifications. Those who participated in the GoNAPSACC program received portable play equipment, which may also explain their interest in fixed play upgrades (e.g., paths). One way to reduce the cost would be to involve parents in the process as volunteers or in other ways, as was suggested by one member check focus groups, and as has been performed by others [28]. In this case, the academic-community partnership used parents to help implement those changes (as manual labor), but purchased the materials from a retailer (~$3K USD) [28]. High quality and sustainable materials will be important, as weather was mentioned as a common barrier by directors and by other studies in ECE settings [50, 51]. Notably, there was no mention of changing policies or systems that may impact outdoor time, which are still areas of improvement as only a slight majority met the best practice of 60+ minutes/day of outdoor time (66.7%), and few met the best practice of offering outdoor time 3+ times/day (11.1%). These changes may have a lower financial cost compared to physical changes to the environment, but changing policies and processes may still require major restructuring to meet safety and capacity standards.

Directors who participated in the interviews were interested in nature-based elements, including gardening and bringing their curriculum outdoors. In anticipation of this interest, the tire garden was included in the pilot study and was well received. Gardens in ECE settings may improve child dietary intake, such as fruit and vegetable consumption, but it is still not clear if a garden influences physical activity [52]. Though most directors were not familiar with the term nature-based, there was excitement about using the outdoors as a classroom. Examinations in Canada have reported that ECE settings increased time outdoors during the COVID-19 pandemic to help with health precautions, and even performed curriculum outdoors [53]. The current study was conducted near the end of the pandemic; thus, directors with recent experiences utilizing this additional outdoor time may be more enthusiastic about this venture. On the other hand, health precautions can also decrease class size and time available outdoors. Still, no director identified a specific academic curriculum with an outdoor accompaniment, only outdoor games that were played.

An unexpected finding was the enthusiasm for the regional GoNAPSACC program, which was a similar format to our pilot study. Both included training on outdoor play from GoNAPSACC curriculum, and incorporated funding for outdoor play upgrades. As expected, many directors did not have formal training in keeping children active outdoors, demonstrating a need for teacher training [15, 16]. Directors mentioned that licensing plays a big role and dictates their current outdoor practices. The state in which data were collected does not have a licensing requirement for teacher training in physical activity, but recently enacted state standards for physical activity [16]. Another U.S. state incorporated a state standard requiring one physical activity training per year, amongst other requirements for physical activity, and this change was related to an improvement in teacher and staff training best practices performed [54]. Directors were interested in in-person training conducted by experts in physical activity. This model is very similar to the technical assistance of other state-wide GoNAPSACC programs [40, 55], which have been effective at improving best practices and outdoor environments in ECE settings.

Strengths of the current study include the critical age of the participants, timely topic, the mixed methods design, and the pragmatic and cost-effective parameters. The semi-structured interviews included a variety of ECE settings. A limitation of the small-scale pilot study was that the participants were predominantly White and high-income. Replication in a larger sample with a randomized design and other populations is warranted to increase the certainty of the evidence. Moreover, this study was confined to the elements presented, and examination of other elements and iterations is needed to support various outdoor settings and toddler interests. Directors’ best practices were also self-reported in both aims; thus, their scores may be higher due to social desirability bias. However, these scores were low in general, and were comparable to other examinations in ECE centers [15, 16]. This study may also be subject to observation bias, as the lead teacher was aware of the program’s purpose and more likely to encourage use of elements during the direct observation sessions. Even so, elements were consistently used in afternoon sessions where the lead teacher was not present, and mainly within groups of children (e.g., walking together on tree cookies), suggesting that there was genuine interest in elements. Because directors who were interested in outdoor time and nature-based elements may have been more likely to participate in this study, we caution that these results may not translate to all ECE settings, and that not all ECE settings may be interested in nature-based elements. Moreover, these results may not translate to other U.S. states, countries, or other ECE settings (e.g., home-based providers) that may have different climate and outdoor environment stipulations or requirements [37, 38]. This study took place during the COVID-19 pandemic, which hindered recruitment for both the pilot and semi-structured interviews, and which could have impacted the barriers and facilitators described.

These results suggest four major future directions for research and practice. First, incorporating policies and systems may bolster the current program, as programs with policy, system, and environment changes are effective and support long-term changes to outdoor time [12]. Second, incorporation of other community partners, such as agriculture departments and continuing education credits, may help facilitate modifications. As discussed by the directors, many facilitated their programs through outside help. Third, a future program may consider integrating and measuring potential changes in cognitive and language skills, which are of importance in this age range. Finally, the effect of this modification on other classrooms (e.g., preschoolers) is not clear, as many classrooms may use the same modified area. Incorporating other classrooms may reduce the teacher burden for continued upkeep.

We found that this pragmatic, cost-effective, nature-based program for toddlers was feasible, and most elements were acceptable. The program had limited effectiveness at improving physical activity, though, as toddlers were already physically active outdoors, as was confirmed by multiple study components. Financial and staffing limitations may hinder major progress to improving outdoor environments for toddlers. Opportunities to provide additional financial support and technical assistance to improve ECE outdoor environments, and an outdoor curriculum, may help support toddlers engaging in nature-based physically active pursuits.

## Supporting information

S1 File (DOCX)

## List of abbreviations

ECE: early childhood education
MVPA: moderate-to-vigorous physical activity
NAPSACC: Nutrition and Physical Activity Self-Assessment for Child-Care Survey
POEMS: Preschool Outdoor Environment Measurement Scale
QUANT: quantitative
QUAL: qualitative
TPA: total physical activity
