# Supplementary material for: Modification of childcare’s outdoor setting for toddler physical activity and nature-based play: A mixed methods study
Source: PLoS One. 2024 Sep 20;19(9):e0309113. doi: 10.1371/journal.pone.0309113 (PMC11414991; doi:10.1371/journal.pone.0309113)
Supplement: S1 File — (DOCX) [file pone.0309113.s001.docx]

| **Supplementary Table 1. Mixed Methods Appraisal Tool** | | |
| --- | --- | --- |
| **Study Design Category** | **Methodological quality criteria** | **Responses** |
| Screening questions  (for all types) | S1. Are there clear research questions? | Introduction, Lines 109-114 |
|  | S2. Do the collected data allow to address the research questions? | Introduction, Lines 105-114 |
| 1. Qualitative | 1.1. Is the qualitative approach appropriate to answer the research question? | Methods, Lines 117-122. |
|  | 1.2. Are the qualitative data collection methods adequate to address the research question? | Methods, Lines 230-241, 274-284 |
|  | 1.3. Are the findings adequately derived from the data? | Results, Lines 349-341, Table 3 and 4 |
|  | 1.4. Is the interpretation of results sufficiently substantiated by data? | Results, Lines 349-341, Table 3 and 4 |
|  | 1.5. Is there coherence between qualitative data sources, collection, analysis, and interpretation? | Results, Lines 463-477, Table 5 |
| 3. Quantitative non-randomized | 3.1. Are the participants representative of the target population? | Methods Lines 117-129, Results, Lines 296-299, Discussion, Lines 561-563 |
|  | 3.2. Are measurements appropriate regarding both the outcome and intervention (or exposure)? | Methods, Lines 132-205 |
|  | 3.3. Are there complete outcome data? | Results, Lines 306-315, 330-347, Supplementary Table 3, Supplementary Figure 2 |
|  | 3.4. Are the confounders accounted for in the design and analysis? | Methods, Lines 132-143 |
|  | 3.5. During the study period, is the intervention administered (or exposure occurred) as intended? | Methods, Lines 206-216, Results, Lines 443-454 |
| 5. Mixed methods | 5.1. Is there an adequate rationale for using a mixed methods design to address the research question? | Introduction, Lines 105-114 |
|  | 5.2. Are the different components of the study effectively integrated to answer the research question? | Results, Lines 463-477, Table 5 |
|  | 5.3. Are the outputs of the integration of qualitative and quantitative components adequately interpreted? | Results, Lines 463-477, Table 5 |
|  | 5.4. Are divergences and inconsistencies between quantitative and qualitative results adequately addressed? | Results, Lines 463-477, Table 5 |
|  | 5.5. Do the different components of the study adhere to the quality criteria of each tradition of the methods involved? | Methods, Lines 105-114, Discussion, Lines 561-563 |

| **Supplementary Table 2. Interview Script for Early Childhood Education Directors on Current Practices on Physical Activity and Outdoor time and Interest in Nature-based and Outdoor Play** | |
| --- | --- |
| **Component** | **Questions** |
| 1. Current Practices related to Toddler Physical Activity and Outdoor Play | - How would you describe your students’ (toddlers in your care) physical activity levels? - What do you view as important influences that could increase toddler’s physical activity levels? Are there factors that prevent toddlers in your care from being more physically active?   - Do these factors differ depending on whether they are outside or inside? - What are some things you do to keep toddlers active while they are inside? - What are some things you do to keep toddlers active while they are outside?   - Do you use fixed structures?   - Do you use portable play items?   - Do you use sports equipment?   - Do you or your teachers participate in the children’s activities while they are outside? - What things do you, or teachers do, to help toddlers be active outside? |
| 2. Interest in Changes to the Outdoor Setting | - If you could change the outdoor environment so toddlers could be more active, what would you do? - Would you be interested in your outdoor play area having more natural options for child play? |
| 3. Barriers and Facilitators to Changing the Outdoor Setting | - What might prevent you from creating nature-based options within these outdoor settings?   - What about for older children (preschoolers)? - What parts of the outdoor environment do you think help toddlers be active during their time outside? - What parts of the outdoor environment make it hard for toddlers to be active? |
| 4. Perspectives on nature-based outdoor play | - Do you have any nature-based options for toddlers to play in within your outdoor setting? |
| 5. Training on Outdoor Play | - What training have you or your teachers received on keeping children active outdoors?   - How many times per year? on what topics? - What types of training would you be interested in for you or your teachers related to outdoor play or outdoor settings? - Who would you like to provide training on outdoor time and outdoor physical activity? |


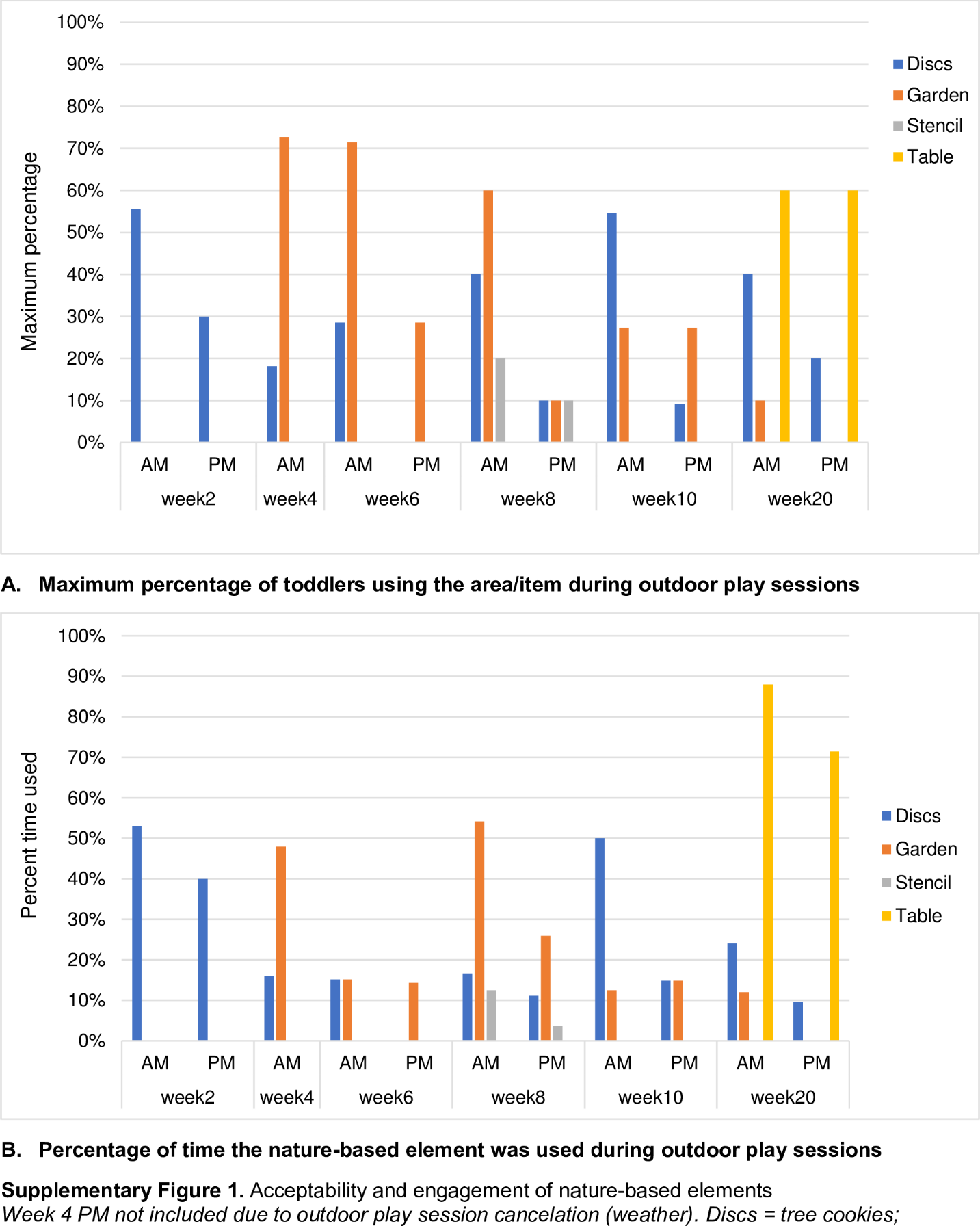


| **Supplementary Table 3. Characteristics and Differences between Groups for Quantitative Study (*n*=15)^** | | | | | | | | | | | | | | | | |  |  |
| --- | --- | --- | --- | --- | --- | --- | --- | --- | --- | --- | --- | --- | --- | --- | --- | --- | --- | --- |
|  |  | **Intervention (n=10)** | | | | **Control (n=5)** | | | | |  | |  | |  | | |  |
|  |  | **Week 0** | | **Week 10** | | **Week 0** | | | **Week 10** | | **Group** | | **Time** | | **Group * Time** | | |  |
|  |  | **Mean ± SD** | **n(%)** | | **Mean ± SD** | | **Mean ± SD** | **n(%)** | | **Mean ± SD** | | ***p*-value** | | ***p*-value** | | ***p*-value** | | |
| Age (months) | | 24.50 ± 2.36 |  | |  | | 31.11 ± 2.57 |  | |  | | 0.001* | |  | |  | | |
| Male | |  | 4(40) | |  | |  | 0 | |  | | 0.23 | |  | |  | | |
| Child Race | |  |  | |  | |  |  | |  | | 0.47 | |  | |  | | |
|  | White |  | 8(80) | |  | |  | 4(80) | |  | | 0.33 | |  | |  | | |
|  | African American |  | 1(10) | |  | |  | 1(20) | |  | |  | |  | |  | | |
|  | Other |  | 1(10) | |  | |  | 0(0) | |  | |  | |  | |  | | |
| Child Hispanic ethnicity | |  | 0 (0) | |  | |  | 1(20) | |  | | 0.33 | |  | |  | | |
| Household Income ($USD) | |  |  | |  | |  |  | |  | | 0.77 | |  | |  | | |
|  | 50,000 - $69,999 |  | 1(10) | |  | |  | 0 | |  | |  | |  | |  | | |
|  | 70,000 - $109,999 |  | 1(10) | |  | |  | 0 | |  | |  | |  | |  | | |
|  | $110,000 or above |  | 8(80) | |  | |  | 4(80) | |  | |  | |  | |  | | |
|  | Prefer not to answer |  | 0 | |  | |  | 1(20) | |  | |  | |  | |  | | |
| *Overall Physical Activity during both outdoor play sessions* | |  |  | |  | |  |  | |  | |  | |  | |  | | |
|  | Outdoor time (minutes) | 66.8 ± 7.9 |  | | 53.0 ± 2.1 | | 81.5 ± 1.0 |  | | 73.4 ± 0.8 | | 0.001* | | 0.001* | | 0.13 | | |
|  | Total PA (minutes) | 62.3 ± 7.3 |  | | 48.9 ± 3.5 | | 75.4 ± 2.3 |  | | 63.9 ± 6.5 | | 0.001* | | 0.001* | | 0.83 | | |
|  | MVPA (minutes) | 30.3 ± 6.5 |  | | 23.2 ± 7.3 | | 28.2 ± 7.8 |  | | 24.5 ± 8.5 | | 0.96 | | 0.15 | | 0.29 | | |
|  | Total PA (%) | 93 ± 2 |  | | 92 ± 4 | | 85 ± 16 |  | | 86 ± 8 | | 0.22 | | 0.20 | | 0.37 | | |
|  | MVPA (%) | 46 ± 9 |  | | 44 ± 15 | | 35 ± 10 |  | | 33 ± 12 | | 0.17 | | 0.79 | | 0.59 | | |
| *Connectedness to Nature and Total Difficulties* | |  |  | |  | |  |  | |  | |  | |  | |  | | |
|  | Awareness of Nature Score (range 1-5)^#^ | 4.55 ± 0.37 |  | | 4.67 ± 0.25 | | 4.08 ± 0.14 |  | | 4.70 ± 0.45 | | 0.30 | | 0.02* | | 0.09 | | |
|  | Empathy for Nature Score (range 1-5) | 3.40 ± 0.75 |  | | 3.67 ± 0.82 | | 4.53 ± 0.69 |  | | 3.93 ± 0.64 | | 0.08 | | 0.36 | | 0.02* | | |
|  | Enjoyment in Nature Score (range 1-5) | 4.47 ± 0.51 |  | | 4.49 ± 0.31 | | 4.47 ± 0.60 |  | | 4.60 ± 0.51 | | 0.81 | | 0.45 | | 0.57 | | |
|  | Responsibility toward Nature Score (range 1-5)^#^ | 3.50 ± 0.89 |  | | 3.63 ± 0.82 | | 3.11 ± 0.51 |  | | 3.00 ± 0.47 | | 0.21 | | 0.93 | | 0.52 | | |
| Total Difficulties (range: 0-40)^#^ | | 9.10 ± 4.48 |  | | 7.78 ± 3.46 | | 11.67 ± 2.52 |  | | 8.60 ± 3.44 | | 0.56 | | 0.12 | | 0.98 | | |
| *^Demographic differences are assessed using an independent t-test or chi-square or fisher exact test, whereas outdoor physical activity during both outdoor play sessions is assessed using a mixed effect model with repeated effects, respectively; PA= physical activity; MVPA = moderate-to-vigorous physical activity; *p<0.05;^#^Missing 2 participants at baseline in the control group, n=3 total, and 1 participant at follow up in the intervention n=9 total;* | | | | | | | | | | | | | | | | |  |  |

**
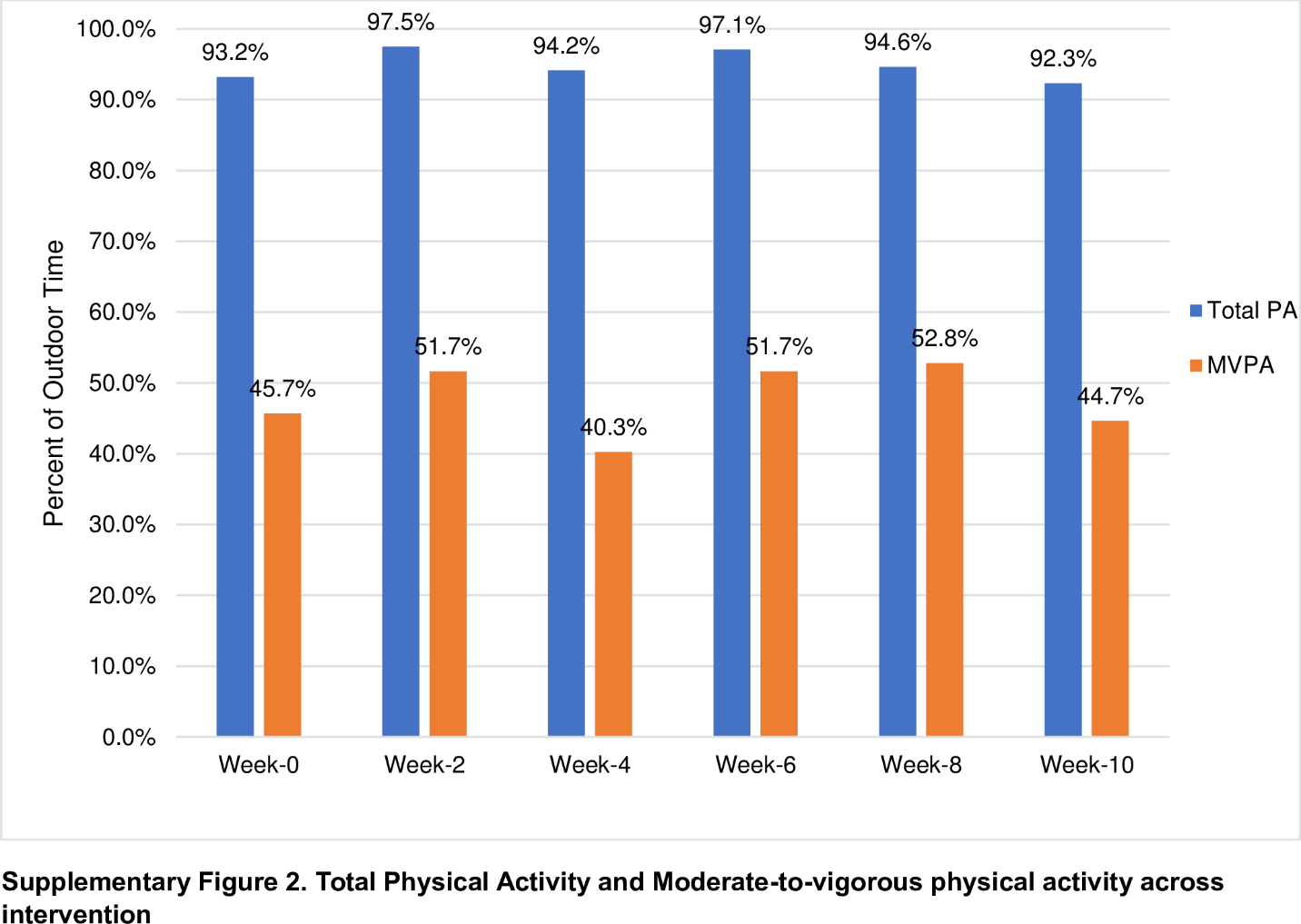
**

**

**
